# Supplementary material for: Genomic and transcriptomic analysis of sacred fig (Ficus religiosa)
Source: BMC Genomics. 2023 Apr 12;24:197. doi: 10.1186/s12864-023-09270-z (PMC10100241; doi:10.1186/s12864-023-09270-z)
Supplement: Supplementary file 1 — Additional file 1: Table S1.1. Details on raw sequence data of F. religiosa genome and transcriptome [file 12864_2023_9270_MOESM1_ESM.docx]

**Genome**

**Supplemental Tables:**

**Table S1.1: Details on raw sequence data of *F. religiosa* genome and transcriptome**

| **Sample (Period of collection)** | **Data type** | **Library type** | **Sequencer** | **Insert length (base)** | **Number of reads (paired-end)** | **Number of bases (paired-end)** |
| --- | --- | --- | --- | --- | --- | --- |
| Leaf | DNA Seq | Paired-end | Illumina HiSeq | 387-390 | 266,593,648 | 23,943,191,355 |
| Leaf | DNA Seq | Paired-end | MGISEQ-2000 | 409-420 | 645,008,220 | 64,500,822,000 |
|  |  |  |  | **Total** | 911,601,868 | 88,444,013,355 |
| Leaf (Day -2 PM) | RNA Seq | Paired-end | Illumina HiSeq | 250-500 | 56,472,586 | 5,613,340,767 |
| Leaf (Night -2 AM) | RNA Seq | Paired-end | Illumina HiSeq | 250-500 | 54,997,562 | 5,469,938,094 |
|  |  |  |  | **Total** | 111,470,148 | 11,083,278,861 |
