# Supplementary material for: Genomic and transcriptomic analysis of sacred fig (Ficus religiosa)
Source: BMC Genomics. 2023 Apr 12;24:197. doi: 10.1186/s12864-023-09270-z (PMC10100241; doi:10.1186/s12864-023-09270-z)
Supplement: Supplementary file 2 — Additional file 2: Figure S1A and S1B. The kmer histogram distribution for the Illumina reads using the GenomeScope tool and The kmer histogram distribution for the MGI reads using the GenomeScope tool [file 12864_2023_9270_MOESM2_ESM.docx]

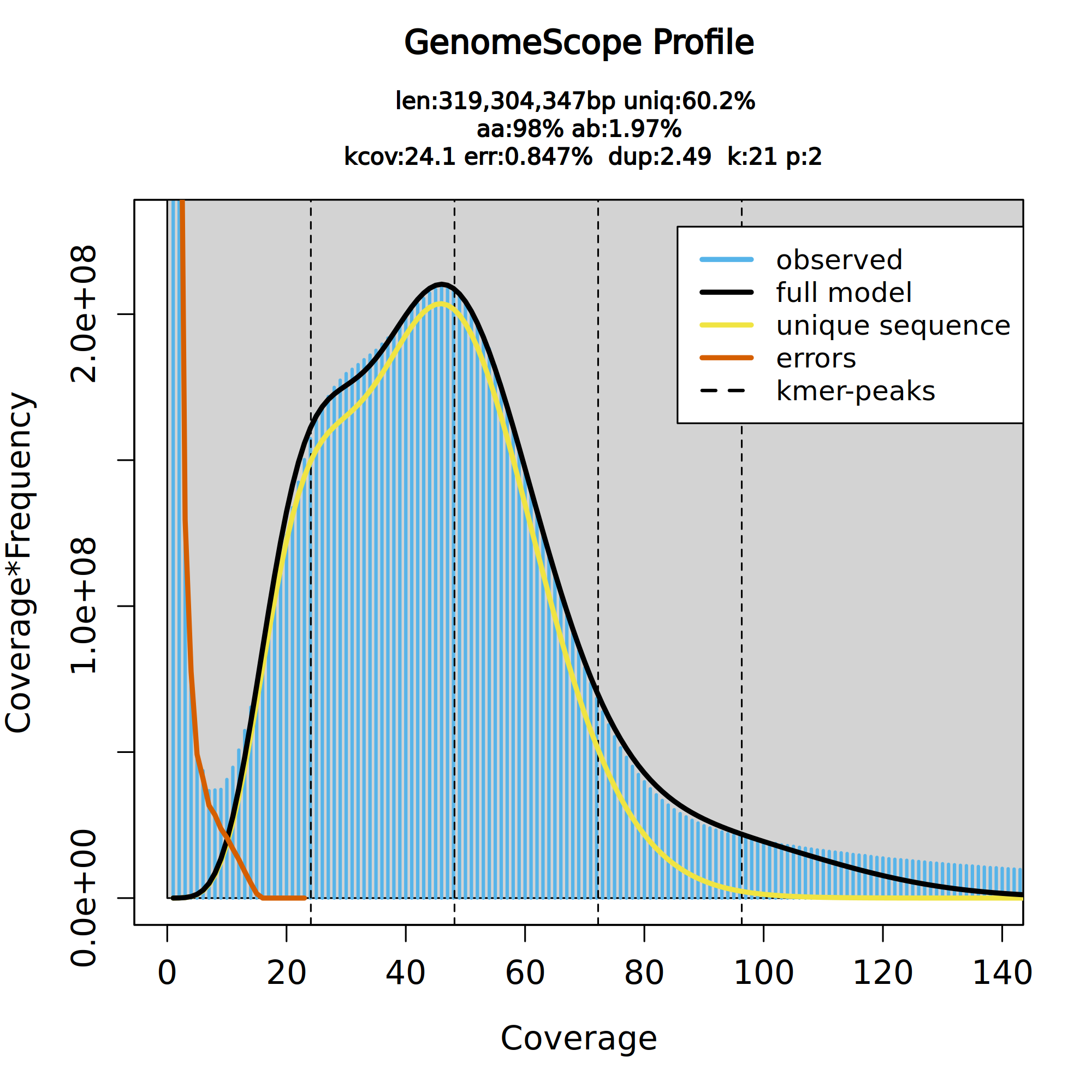


**Figure S1A: The kmer histogram distribution for the Illumina reads using the GenomeScope tool**.


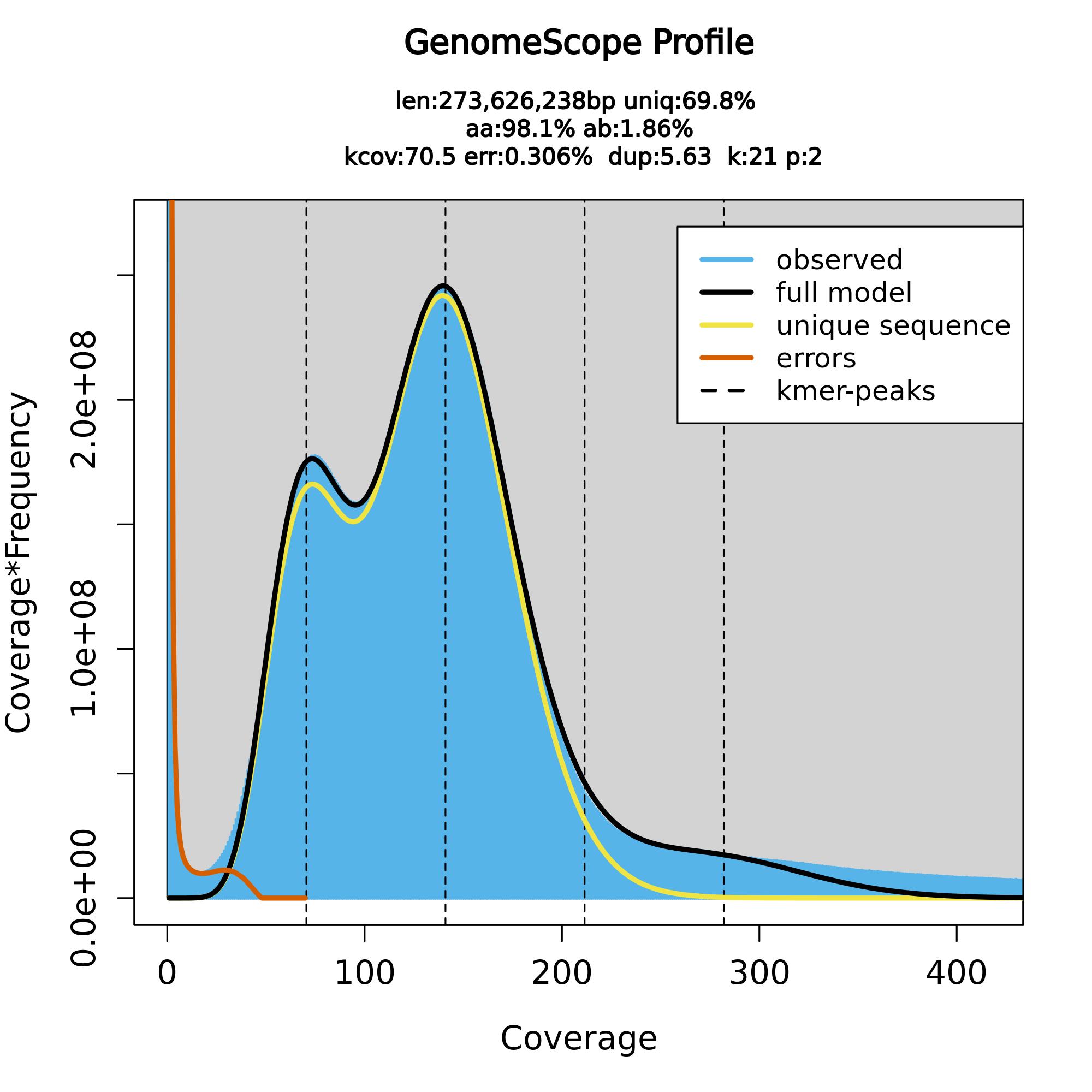


**Figure S1B: The kmer histogram distribution for the MGI reads using the GenomeScope tool.**
