# Supplementary material for: Genomic and transcriptomic analysis of sacred fig (Ficus religiosa)
Source: BMC Genomics. 2023 Apr 12;24:197. doi: 10.1186/s12864-023-09270-z (PMC10100241; doi:10.1186/s12864-023-09270-z)
Supplement: Supplementary file 3 — Additional file 3: Figures S2A. Flow chart of De Novo Whole Genome Analysis (WGA) of Ficus religiosa [file 12864_2023_9270_MOESM3_ESM.docx]

**Figure S2:** Flow chart of *De Novo* Whole Genome Analysis (WGA) of *Ficus religiosa*

Illumina Raw reads

BGI Raw reads

Combined Illumina and BGI

Raw reads

FastQC Quality assesment

Trimgalore Filtering reads

SPAdes Contig assembly

MaSurca Contig assembly

SPAdes Hybrid contig

assembly

SSPACE Super scaffolding

SSPACE Super scaffolding

SSPACE Super scaffolding

QUAST Assembly statistics

GMCloser Gap closing

BUSCO Assembly evaluation

MAKER – P Genome

annotation
