# Supplementary material for: Genomic and transcriptomic analysis of sacred fig (Ficus religiosa)
Source: BMC Genomics. 2023 Apr 12;24:197. doi: 10.1186/s12864-023-09270-z (PMC10100241; doi:10.1186/s12864-023-09270-z)
Supplement: Supplementary file 4 — Additional file 4: Table S1.2. Contig and scaffold assembly statistics of F. religiosa genome [file 12864_2023_9270_MOESM4_ESM.docx]

**Table S1.2: Contig and scaffold assembly statistics of *F. religiosa* genome**

| **Contig assembly** | **Illumina assembly** | **MGI assembly** | **Hybrid assembly** |  |
| --- | --- | --- | --- | --- |
| No. of contigs | 92,794 | 234,282 | 136,974 |  |
| Largest contig (bp) | 45,835 | 29,929 | 95,945 |  |
| Total assembled bases (bp) | 248,292,704 | 300,267,781 | 307,589,416 |  |
| Minimum scaffold length (bp) | 56 | 300 | 78 |  |
| Maximum scaffold length (bp) | 39,390 | 29,929 | 95,945 |  |
| GC content (%) | 33.13 | 33.40 | 33.61 |  |
| N50 (bp) | 4,862 | 1,481 | 3,916 |  |
| L50 | 14,285 | 54,768 | 19,679 |  |
| **Scaffold assembly** | **Illumina assembly** | **MGI assembly** | **Hybrid assembly** | **Gap closed assembly** |
| No. of scaffolds | 87,060 | 118,027 | 121,895 | 121,696 |
| Largest scaffold (bp) | 56,337 | 54,685 | 174,746 | 174,006 |
| Total assembled bases (bp) | 264,662,650 | 345,020,555 | 386,980,121 | 381,047,120 |
| Minimum scaffold length (bp) | 56 | 300 | 78 | 78 |
| Maximum scaffold length (bp) | 56,337 | 54,685 | 174,748 | 174,748 |
| GC content (%) | 32.99 | 33.44 | 33.69 | 33.70 |
| N50 (bp) | 5,340 | 4,771 | 6,482 | 6,385 |
| L50 | 13,703 | 20,196 | 15,619 | 15,539 |
