# Supplementary material for: Genomic and transcriptomic analysis of sacred fig (Ficus religiosa)
Source: BMC Genomics. 2023 Apr 12;24:197. doi: 10.1186/s12864-023-09270-z (PMC10100241; doi:10.1186/s12864-023-09270-z)
Supplement: Supplementary file 6 — Additional file 6: Figures S3A and S3B. BUSCO Assessment results using the plant universal single-copy orthologs (Embryophyta database) and BUSCO Assessment results using the eukaryote universal single-copy orthologs (Eukaryota database). [file 12864_2023_9270_MOESM6_ESM.docx]

**Figure S3A: BUSCO Assessment results using the plant universal single-copy orthologs (embryophyta database)**

**Figure S3B: BUSCO Assessment results using the eukaryote universal single-copy orthologs (eukaryota database)**
