# Supplementary material for: Genomic and transcriptomic analysis of sacred fig (Ficus religiosa)
Source: BMC Genomics. 2023 Apr 12;24:197. doi: 10.1186/s12864-023-09270-z (PMC10100241; doi:10.1186/s12864-023-09270-z)
Supplement: Supplementary file 10 — Additional file 10: Table S2. Top 10 pathways with the highest gene counts in the F. religiosa genome [file 12864_2023_9270_MOESM10_ESM.docx]

**Table S2: Top 10 pathways with highest gene counts in *F. religiosa* genome**

| **Pathway names** | **Gene counts** |
| --- | --- |
| Ribosome | 123 |
| Spliceosome | 96 |
| Oxidative phosphorylation | 86 |
| Thermogenesis | 82 |
| RNA transport | 74 |
| Protein processing in endoplasmic reticulum | 73 |
| Huntington disease | 68 |
| Parkinson disease | 57 |
| Endocytosis | 55 |
| Alzheimer disease | 55 |
