# Supplementary material for: Genomic and transcriptomic analysis of sacred fig (Ficus religiosa)
Source: BMC Genomics. 2023 Apr 12;24:197. doi: 10.1186/s12864-023-09270-z (PMC10100241; doi:10.1186/s12864-023-09270-z)
Supplement: Supplementary file 12 — Additional file 12: Figure S4. Phylogenetic analysis of Ficus religiosa with other plant species like A. thaliana, C. sativa, M. notabilis, Z. jujuba, P. persica [file 12864_2023_9270_MOESM12_ESM.docx]

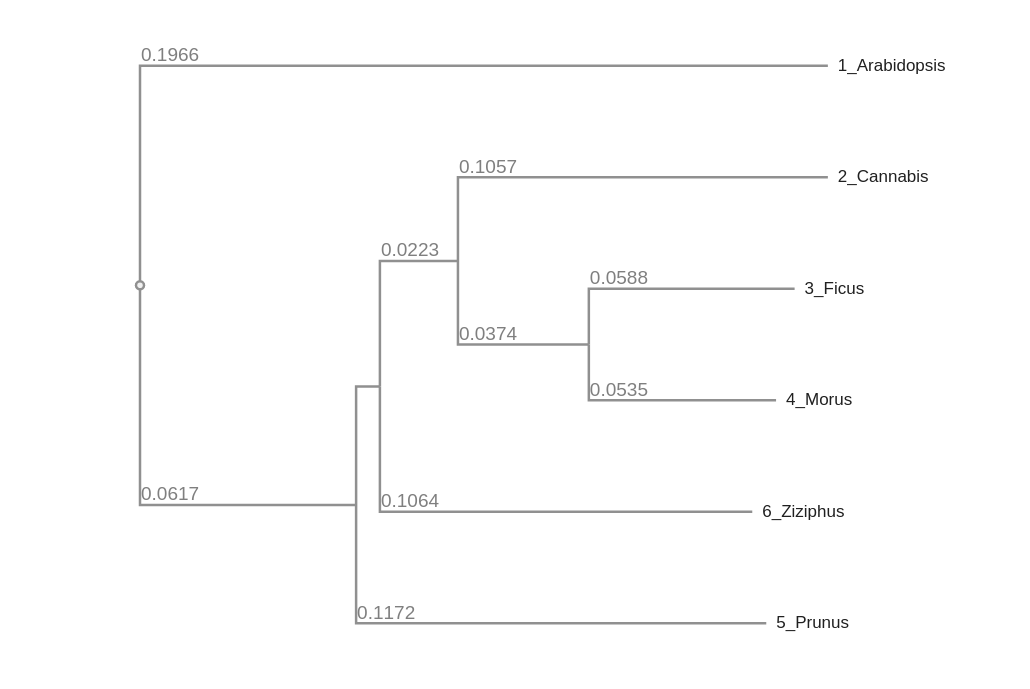


**Figure S4: Phylogenetic analysis of *Ficus religiosa* with other plant species like *A. thaliana, C. sativa*, *M. notabilis, Z. jujuba, P. persica***
