# Supplementary material for: Genomic and transcriptomic analysis of sacred fig (Ficus religiosa)
Source: BMC Genomics. 2023 Apr 12;24:197. doi: 10.1186/s12864-023-09270-z (PMC10100241; doi:10.1186/s12864-023-09270-z)
Supplement: Supplementary file 14 — Additional file 14: Table S4. Repeat content in the assembled F. religiosa genome [file 12864_2023_9270_MOESM14_ESM.docx]

**Table S4: Repeat content in the assembled *F. religiosa* genome**

| **Classification** | **Copy number** | **DNA content (nts)** | **DNA content (%)** |
| --- | --- | --- | --- |
| **Non-LTR Retrotransposon** |  |  |  |
| LINE1 | 2,723 | 939,296 | 0.19 |
| LTR-Retrotransposon | 134,518 | 25,612,270 | 5.09 |
| DNA transposons | 25,332 | 5,494,527 | 1.09 |
| Unclassified | 1,805,148 | 220,080,246 | 43.71 |
| Interspersed repeats | - | 252,126,339 | 50.07 |
| Simple Sequence repeats (SSR) | 368,277 | 16,386,007 | 3.25 |
| Low complexity | 45,966 | 2,238,964 | 0.44 |
| **Total repeat size** |  | **269.62 Mb** | **53.55 %** |
