# Supplementary material for: Genomic and transcriptomic analysis of sacred fig (Ficus religiosa)
Source: BMC Genomics. 2023 Apr 12;24:197. doi: 10.1186/s12864-023-09270-z (PMC10100241; doi:10.1186/s12864-023-09270-z)
Supplement: Supplementary file 15 — Additional file 15: Table S5.1. Simple sequence repeats (SSR) prediction in the genome of F. religiosa [file 12864_2023_9270_MOESM15_ESM.docx]

**Table S5.1: Simple sequence repeats (SSR) prediction in the genome of *F. religiosa***

| **Features** | **Numbers** |
| --- | --- |
| No. of sequences | 1,189,520 |
| Total size of sequences (bp) | 503,523,744 |
| Total no. of identified SSRs | 799,992 |
| No. SSR containing sequences | 267,593 |
| Mono repeats | 606,169 |
| Di repeats | 143,113 |
| Tri repeats | 34,327 |
| Tetra repeats | 11,791 |
| Penta repeats | 2,911 |
| Hexa repeats | 1,681 |
