# Supplementary material for: Genomic and transcriptomic analysis of sacred fig (Ficus religiosa)
Source: BMC Genomics. 2023 Apr 12;24:197. doi: 10.1186/s12864-023-09270-z (PMC10100241; doi:10.1186/s12864-023-09270-z)
Supplement: Supplementary file 19 — Additional file 19: Figure S5. Flow chart of De novo Transcriptome Analysis of Ficus religiosa. [file 12864_2023_9270_MOESM19_ESM.docx]

**Figure S5:** Flow chart of *De novo* Transcriptome Analysis of *Ficus religiosa*

Raw reads

Trinity *De novo* assembly

Transdecoder Coding sequence prediction

NR db: BLASTX

Uniprot db: BLASTP

EdgeR Differential gene expression analysis

RSEM Transcript quantification and estimation

CDHIT-est Set of non-redundant transcripts

Trinotate Annotation

Pfam Protein families

GO Term Annotation

Blast2Go Annotation
