# Supplementary material for: Genomic and transcriptomic analysis of sacred fig (Ficus religiosa)
Source: BMC Genomics. 2023 Apr 12;24:197. doi: 10.1186/s12864-023-09270-z (PMC10100241; doi:10.1186/s12864-023-09270-z)
Supplement: Supplementary file 20 — Additional file 20: Table S7. Assembly Statistics of F. religiosa Transcriptome [file 12864_2023_9270_MOESM20_ESM.docx]

**Table S7: Assembly Statistics of *F. religiosa* Transcriptome**

| **Counts of transcripts** | **Assembly (Day - 2PM)** | **Assembly (Night -2AM)** | **Combined Assembly** |
| --- | --- | --- | --- |
| Number of genes | 35,928 | 36,271 | 43,227 |
| Number of transcripts | 82,679 | 90,498 | 116,038 |
| GC content (%) | 42.75 | 42.42 | 42.17 |
| Contig N50 (bp) | 1,914 | 2,053 | 2,076 |
| Median contig length (bp) | 929 | 1,011 | 969 |
| Average Contig (bp) | 1238.49 | 1332.73 | 1316.83 |
| Total assembled bases (bp) | 102,397,092 | 120,608,982 | 152,802,655 |
