# Supplementary material for: Genomic and transcriptomic analysis of sacred fig (Ficus religiosa)
Source: BMC Genomics. 2023 Apr 12;24:197. doi: 10.1186/s12864-023-09270-z (PMC10100241; doi:10.1186/s12864-023-09270-z)
Supplement: Supplementary file 25 — Additional file 25: Table S10.1. Details on long non-coding RNAs, miRNAs in De novo transcripts, reference-based transcripts, and genome [file 12864_2023_9270_MOESM25_ESM.docx]

**Table S10.1: Details on long non-coding RNAs, miRNAs in *De novo* transcripts, reference-based transcripts and genome**

| **Genes** | **Total transcript / contig** | **No. of coding transcripts** | **No. of non-coding transcripts** | **Non-coding transcripts v/s genome annotated genes (BLASTN)** | **miRNAs families (micro RNAs) predicted** | **Homologous miRNA in other organisms** | **No. of transcripts consists miRNAs** |
| --- | --- | --- | --- | --- | --- | --- | --- |
| *De novo* transcripts | 26,691 | 19,911 | 6,780 | 4,219 | MIR 168 and its subfamilies | mdm, ppe, tcc, mes, crt, csi, ccl, gma, mtr, fve, vun, aau, lja, cme, vvi, ptc, nta, lus, bra, aof, nta, rco, cln, pta, far | 2 |
| Reference based transcript | 30,973 | 7,163 | 23,810 | 14,605 | MIR396, MIR7534, MIR166, MIR6236, MIR399, MIR168, MIR4995, MIR2916, MIR171, MIR3629, MIR5523, MIR8576, MIR6173, MIR156, MIR393, MIR827, MIR172, MIR167, MIR529, MIR394, MIR398, MIR408, MIR5538, MIR162, | csi, fve, gma, ccl, ptc, sly, atr, tcc, mes, nta, pab, ppe, vvi, cme, mdm, lja, cln, pta, mmu, crt, mtr, vun, aau, lus, bra, aof, aly, ath, rco, peu, bdi, bcy, bgy, hbr, amg, zma, sof, sbi, ghr, cas, osa, eun, ata, vca, ctr, bna, pab, egu, dpr, cca, far, cpa, hpe, htu | 30 |
| Day sample transcript | 27,156 | 6,628 | 20,528 | 18,893 | MIR7534, MIR166, MIR6236, MIR168, MIR4995, MIR2916, MIR11602, MIR171, MIR396, MIR3629, MIR5523, MIR8576, MIR6173, MIR156, MIR393, MIR827, MIR399, MIR167, MIR529, MIR398, MIR5141, MIR5538, MIR162 | lja, cln, pta, mmu, mdm, ppe, tcc, mes, crt, csi, ccl, gma, mtr, fve, vun, aau, cme, vvi, ptc, nta, lus, bra, aof, aly, ath, rco, peu, pla, atr, bdi, bcy, bgy, hbr, amg, aau, lus, zma, sof, sbi, sly, ghr, cas, osa, eun, ata, vca, ppe, aof, dpr, cca, far, cas, ahy, gra, rgl, cpa, vun, hpe, htu | 26 |
| Night sample transcript | 32,833 | 7,339 | 25,494 | 22,232 | MIR396, MIR166, MIR6236, MIR399, MIR168, MIR4995, MIR11602, MIR5523, MIR6173, MIR827, MIR167, MIR5538, MIR5141, MIR408, MIR7534, MIR2916, MIR171, MIR3629, MIR8576, MIR156, MIR393, MIR398, MIR162 and their subfamilies | csi, fve, gma, ccl, ptc, sly, atr, tcc, mes, nta, pab, ppe, vvi, cme, mdm, cln, pta, mmu, crt, mtr, vun, aau, lja, lus, bra, aof, aly, ath, rco, pla, bdi, bcy, bgy, hbr, amg, zma, sof, sbi, ghr, cas, osa, eun, ata, vca, rgl, peu, hbr, dpr, cca, far, cas, ahy, gra, cpa, hpe, htu | 31 |
| Genome | 121,696 | - | - | - | MIR2916, MIR8175, MIR6236, MIR396, MIR7534, MIR166, MIR156, MIR157, MIR164, MIR395, MIR399, MIR319, MIR297, MIR529, MIR168, MIR4995, MIR5538, MIR11602, MIR171, MIR172, MIR160, MIR169, MIR396, MIR8576, MIR3629, MIR2111, MIR390, MIR6300, MIR6236, MIR5523, MIR167, MIR170, MIR6173, MIR5141, MIR5368, MIR159, MIR393, MIR6478, MIR8005, MIR827, MIR394, MIR398, MIR397, MIR408, MIR828, MIR477 and their subfamilies | peu, ath, mmu, csi, fve, gma, ccl, ptc, sly, atr, tcc, mes, nta, pab, ppe, vvi, cme, mdm, lja, cln, pta, hbr, aof, aqc, rco, vun, bcy, bgy, lus, vca, gra, mtr, stu, cpa, cas, bna, aly, ctr, rgl, ssl, eun, nta, dpr, bdi, pta, osa, mml, mdm, ahy, crt, aau, bra, hbr, atr, stu, pla, cca, pvu, ghb, amg, zma, sof, sbi, ghr, htu, hpa, ssl, egu, far, | 429 |
