# Supplementary material for: Genomic and transcriptomic analysis of sacred fig (Ficus religiosa)
Source: BMC Genomics. 2023 Apr 12;24:197. doi: 10.1186/s12864-023-09270-z (PMC10100241; doi:10.1186/s12864-023-09270-z)
Supplement: Supplementary file 26 — Additional file 26: Table S10.2. Statistics of Transfer RNAs predicted in the genome [file 12864_2023_9270_MOESM26_ESM.docx]

**Table S10.2: Statistics of Transfer RNAs predicted in the genome**

| **Features** | **Numbers** |
| --- | --- |
| No. of bases in tRNAs | 83,569 |
| Total tRNAs predicted | 1,104 |
| Average tRNA length | 75 |
| Infernal-confirmed tRNAs | 1,046 |
| tRNAs decoding standard 20 amino acids | 890 |
| Suppressor tRNAs | 2 |
| tRNAs with unknown isotypes | 4 |
| Predicted pseudogenes | 150 |
| tRNAs with introns | 59 |
