# Supplementary material for: Genomic and transcriptomic analysis of sacred fig (Ficus religiosa)
Source: BMC Genomics. 2023 Apr 12;24:197. doi: 10.1186/s12864-023-09270-z (PMC10100241; doi:10.1186/s12864-023-09270-z)
Supplement: Supplementary file 28 — Additional file 28: Figure S6. Graphical representation of differentially expressed genes of Ficus religiosa mapped to reference Carbon fixation in photosynthetic organisms pathway (map00710) from KEGG. [file 12864_2023_9270_MOESM28_ESM.docx]

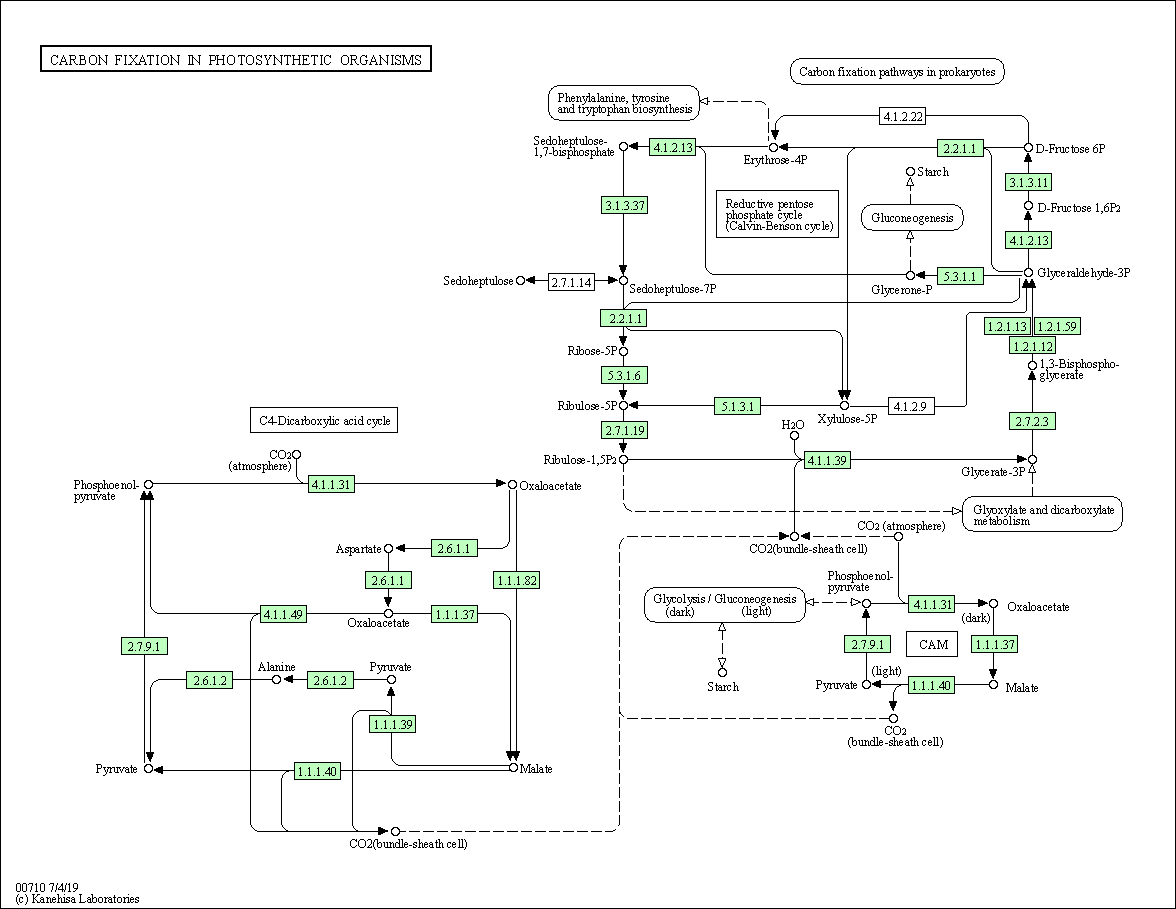


**Figure S6: Graphical representation of differentially expressed genes of *Ficus religiosa* mapped to reference Carbon fixation in photosynthetic organisms pathway (map00710) from KEGG. The green color boxes indicate *F. religiosa* genes mapping on the reference and the white boxes indicate no mapping.**

**Source: The permission to use of KEGG pathway image is granted under the CC BY 4.0 open-access license from Kanehisa Laboratories and sources are listed in the references.**
