# Supplementary material for: Genomic and transcriptomic analysis of sacred fig (Ficus religiosa)
Source: BMC Genomics. 2023 Apr 12;24:197. doi: 10.1186/s12864-023-09270-z (PMC10100241; doi:10.1186/s12864-023-09270-z)
Supplement: Supplementary file 29 — Additional file 29: Figure S7. Diagrammatic representation of candidate genes of Carbon fixation pathway [file 12864_2023_9270_MOESM29_ESM.docx]

**Figure S7: Diagrammatic representation of candidate genes of Carbon fixation pathway (C3, C4, CAM cycle)**
